# Programming of neurotoxic cofactor CXCL-10 in HIV-1-associated dementia: abrogation of CXCL-10-induced neuro-glial toxicity in vitro by PKC activator

**DOI:** 10.1186/1742-2094-9-239

**Published:** 2012-10-18

**Authors:** Rajeev Mehla, Shalmali Bivalkar-Mehla, Mitzi Nagarkatti, Ashok Chauhan

**Affiliations:** 1 Department of Pathology, Microbiology & Immunology, University of South Carolina, School of Medicine, Columbia, SC, 29209, USA; 2 Department of Pharmacology, Physiology and Neuroscience, University of South Carolina, Columbia, SC, 29209, USA

**Keywords:** TNF-α, CXCR4/CXCR3, Cytokines, Chemokines, Chemotaxis, Bryostatin

## Abstract

**Background:**

More than 50% of patients undergoing lifelong suppressive antiviral treatment for HIV-1 infection develop minor HIV-1-associated neurocognitive disorders. Neurological complications during HIV-1 infection are the result of direct neuronal damage by proinflammatory products released from HIV-1-infected or -uninfected activated lymphocytes, monocytes, macrophages, microglia and astrocytes. The specific pro-inflammatory products and their roles in neurotoxicity are far from clear. We investigated proinflammatory cytokines and chemokines in the cerebrospinal fluid (CSF) of HIV-demented (HIV-D) and HIV-nondemented (HIV-ND) patients and studied their affect on neuroglial toxicity.

**Methods and results:**

Bioplex array showed elevated levels of signatory chemokines or cytokines (IL-6, IFN-γ, CXCL10, MCP-1 and PDGF) in the CSF of HIV-D patients (n = 7) but not in that of HIV-ND patients (n = 7). Among the signatory cytokines and chemokines, CXCL10 was distinctly upregulated in-vitro in HIV-1 (NLENG1)-activated human fetal astrocytes, HIV-1 (Ba-L)-infected macrophages, and HIV-1 (NLENG1)-infected lymphocytes. Virus-infected macrophages also had increased levels of TNF-α. Consistently, human fetal astrocytes treated with HIV-1 and TNF-α induced the signatory molecules. CXCL10 in combination with HIV-1 synergistically enhanced neuronal toxicity and showed chemotactic activity (~ 40 fold) for activated peripheral blood mononuclear cells (PBMC), suggesting the intersection of signaling events imparted by HIV-1 and CXCL10 after binding to their respective surface receptors, CXCR4 and CXCR3, on neurons. Blocking CXCR3 and its downstream MAP kinase (MAPK) signaling pathway suppressed combined CXCL10 and HIV-1-induced neurotoxicity. Bryostatin, a PKC modulator and suppressor of CXCR4, conferred neuroprotection against combined insult with HIV-1 and CXCL10. Bryostatin also suppressed HIV-1 and CXCL10-induced PBMC chemotaxis. Although, therapeutic targeting of chemokines in brain may have adverse consequences on the host, current findings and earlier evidence suggest that CXCL10 could strongly impede neuroinflammation.

**Conclusion:**

We have demonstrated induction of CXCL10 and other chemokines/cytokines during HIV-1 infection in the brain, as well as synergism of CXCL10 with HIV-1 in neuronal toxicity, which was dampened by bryostatin.

## Introduction

HIV-1 infection programs aberrant host immune responses, including those in the brain that contribute to neuropathogenesis [1]. HIV infection in the brain is characterized by the formation of multinucleated giant cells (MNGCs), microglial nodules, and astrocytosis, which result in neuronal damage and loss of dendrites. About 25% of untreated patients with chronic HIV-1 infection develop HIV-associated dementia (HAD). More than 50% of successfully treated patients develop mild neurological deficits known as HIV-1-associated neurocognitive disorders. Unfolding the mechanism of neuronal damage can lead to the identification of new therapeutic drug targets. M-tropic HIV-1 infects macrophages and microglia in the brain, but both M-tropic and T-tropic HIV-1 populations were also found on autopsy of central nervous tissues and cerebrospinal fluid (CSF) of HAD patients [2-5]. HIV-1-derived proteins, including envelope, Tat, Nef, and Vpr cause direct neuronal damage. In addition, neurotoxic inflammatory products have been implicated in neuronal damage [6-10]. These products include proinflammatory cytokines and chemokines derived from HIV-1 infected- or uninfected- activated lymphocytes, macrophages, microglia, and astrocytes.

TNF-α is a monocyte-derived proinflammatory cytokine [11]. Its immunoreactivity has been reported in frontal white matter from the autopsied brains of patients with HIV-1 infection [12]. During HIV-1 infection in the brain, activated macrophage- and microglia-secreted TNF-α acts in an autocrine and paracrine manner that, in combination with viral proteins, amplifies immunological and inflammatory responses in the central nervous system (CNS) [13-17]. Membrane-bound TNF-α on monocyte-derived macrophages (MDM) causes astrocyte toxicity by cell-to-cell contact [18]. Overexpression of TNF-α *in vivo* causes T-cell infiltration in the CNS, as well as astrocytosis, microgliosis, and demyelination [19]. Astrocytes respond to TNF-α with increases in CXCR3, CCR3, and CXCR1 receptors [20], as well as CXCL10 [21], resulting in transduction of downstream signals via binding of cytokine or chemokine ligands.

CXCL10, a 10-kDa IFN-γ-induced protein (IP-10) belonging to the CXC chemokine family, is secreted in response to IFN-γ from different cells, including monocytes, endothelial cells, fibroblasts, astrocytes, and lymphocytes [22,23]. CXCL10 elicits its effects by binding to chemokine receptor CXCR3 [24]. Elevated levels of CXCL10 correlate with the development of HIV-D [25] and are associated with other CNS disorders [26-31].

HIV proteins such as gp120, Nef, and Tat induce CXCL10 [32-34]. A few studies have shown a correlation between elevated levels of CXCL10 in the CSF and plasma of individuals with HIV-1 infection and the association of CXCL10 with the severity of clinical symptoms [9,25,35-37]. In addition, brain tissues from individuals with HAD have been shown to have elevated mRNA levels of CXCL10 [32]. Chronic expression of monocyte chemotactic protein (MCP)-1 and CXCL10 in HIV-1-infected brains increases the migration of monocytes and CXCR3+ T-cells into the brain [37-40].

Current antiretroviral therapy has significantly reduced the severity of HIV-1 and neurological complications. However, residual viral activity, consequent neuroinflammation, and immune activation continue to account for neurological complications [41]. Lack of anti-inflammatory and HIV-1 transcriptional repressors in current antiviral therapy require a search for new anti-inflammatory and neuroprotective agents. Bryostatin, a macrocyclic lactone isolated from endosymbiont γ-proteobacterial *Endobugula sertula*[42], is an activator of protein kinase C (PKC) and has been shown to have diverse biological activities. Recently, we and others have shown that bryostatin has anti-HIV-1 activity via various mechanisms, such as blocking the effect of stromal-cell-derived factor-1 (SDF-1), which it does by down regulating its CXCR4 receptors [43] or modulating CD4 and CXCR4 receptors [44,45]. Bryostatin is currently being used in a phase I clinical trial in patients with nonhematologic tumors, and in a phase II trial for relapsed multiple myeloma [46,47]. In the brain, bryostatin specifically activates PKC epsilon, resulting in neurite extension and the development of long-term memory and neuroprotection [48-50].

Although several studies have been published on proinflammatory products in HIV-1-demented patients, no guidelines are available for selective targeting of proinflammatory molecules. In the current study, we investigated the proinflammatory response in the CSF of HIV-1-infected demented and nondemented patients. We further investigated whether HIV-1 infection (M-tropic) in macrophages or treatment of astrocytes with TNF-α and HIV-1 (T-tropic or M-tropic) provoked the same proinflammatory response as that in patients. We have shown that HIV-1 infection in macrophages or treatment of astrocytes with TNF-α and HIV-1 modulates CXCL10 expression, which leads to neuro-toxicity. We further investigated whether bryostatin, a PKC activator, confers neuroprotection against combined HIV-1 and CXCL10 insult.

## Materials and methods

### Cerebrospinal fluid samples

CSF samples from seven HIV-1-infected demented (HIV-D) patients and seven HIV-1-infected nondemented (HIV-ND) patients were obtained through the National NeuroAIDS Tissue Consortium (NNTC, Rockville, MD, USA) and were IRB exempt. The ages of HIV-D patients ranged from 33 to 52 years, with a median age of 41 years; that of HIV-ND patients ranged from 36 to 50 years, with a median of 39 years. Six HIV-D patients were African-American males; one was female, with viral load ranging from 50 to 512. All HIV-ND patients were white and male, with undetectable viral load except for two patients with 69 and 19 viral copies respectively. Before testing, CSF samples were thawed, spun briefly to remove the precipitated proteins, and diluted 1:2 with optiMEM. The diluted samples were tested in a custom made ten-plex array (Bio-Rad).

### Primary human brain cultures, peripheral blood mononuclear cells (PBMCs), and cell lines

Primary human brains at 8 to 12 weeks gestational age obtained from the University of Washington, Seattle were cultured as described earlier [51,52]. Briefly, meninges and blood vessels were removed from the brain cortex. The tissue was washed twice with optiMEM containing 1% penicillin-streptomycin and amphotericin-B (GibcoBRL). The cortex tissues were mechanically disrupted by passing them once through a 20-ml syringe without a needle. Disrupted cortex tissue was centrifuged at 1,200 rpm for 20 minutes at 4°C. For differentiation to astrocytes, cell pellets were resuspended in DMEM supplemented with antibiotics and 10% FCS negative for *Mycoplasma sps*. For differentiation to neurons, pellets were resuspended in opti-MEM medium and cultured in Opti-MEM supplemented with 5% FCS, 0.2% N2 supplement (GibcoBRL), and antibiotics. The purity of cultures was established by immunostaining either microtubule-associated protein-2 (Sigma) for neurons or glial fibrillary acidic protein (GFAP) for astrocytes.

Primary human fetal neuron (HFN) and astrocyte (HFA) cultures were maintained for at least a month before use. Astrocytes were used at the second or third passage to discourage growth of contaminating microglia. Cultures derived from human fetal brain tissues less than 14 weeks of age lack microglia. The cultures were verified for purity by immunostaining using GFAP, MAP 2, and CD68 markers. Astrocyte cultures were more than 95% pure with no evidence of microglia; neurons were observed, but rarely [52,53].

PBMCs were separated by Histopaque (Sigma), washed three times with PBS, and seeded in RPMI medium supplemented with 10% fetal bovine serum (FBS) and 10% human AB plasma [54]. Monocytes were removed by selective adherence to plastic and differentiated into macrophages for 5 to 7 days without cytokines [54].

Jurkat, 293T, and Magi cells were obtained from the National Institutes of Health (NIH) AIDS Research and Reference Reagent Program, Division of AIDS, NIAID. Jurkat and 293T cells were maintained in RPMI-1640, and Magi cells in DMEM with 2 mM L-glutamine supplemented with 10% FBS and 1% each of penicillin, streptomycin, and amphotericin-B (GibcoBRL). All cultures, primary cells and cell lines were tested for absence of *Mycoplasma sps* by PCR using primers from Stratagene.

### Virus packaging and infection

Full-length T-tropic HIV-1 NL4-3 [55], HIV-1 NLENG1 (NL4-3 viral DNA expressing GFP [52,56] and mutant HIV-1 NL4-3 (D116N) and D64A defective in their integrase function [57] were used as controls [52,56]. Virus particles were packaged in 293T cells. Briefly, 17 μg of HIV-1 expressing plasmids were transfected in 100-mm culture dishes (BD Falcon) using Lipofectamine 2000 (Invitrogen), as published earlier [52]. The supernatants containing viral particles were harvested 72 h after transfection and centrifuged at 300g for 15 minutes at 4°C to remove cellular debris. They were treated with 5 IU/ml RNase-free DNase (Qiagen) for 15 minutes at room temperature (RT) and filtered through 0.22 μm membrane filter. Aliquots of 1.0 ml were stored at -80°C; viral titers were determined by p24 ELISA (Zeptometrix) or infection of Jurkat cells, as reported earlier [45]. Human MDM were infected using M-tropic HIV-1 Ba-L strain (100 ng/ml p24) for 2 h and washed twice with medium; the infection was monitored for up to 21 days. Peak viral activity was detected by p24 assay (ELISA) between 12 and 16 days after infection. Harvested supernatants were analyzed for chemokines and cytokines, and eventually for neurotoxic activity. Jurkat cells were infected with NLENG1 (50 ng/ml p24) for 2 h, washed twice with medium, and observed for 7 days. Cultures were monitored for green fluorescent protein (GFP) expression and supernatants for viral p24.

### Bioplex array and ELISA

Using a custom-made ten-plex Bioplex array (CXCL10, IFN-γ, IL-1β, IL-6, IL-8, IL-17, MCP-1, macrophage inflammatory protein (MIP)-1α, platelet-derived growth factor (PDGF)-bb, TNF-α) according to the manufacturer’s protocol (Bio-Rad), we analyzed cytokine and chemokine in CSF samples (diluted 1:2 in optiMEM) from seven HIV-ND patients and seven HIV-D patients, as well as in supernatants from HIV-1-infected macrophages at 16 days after infection, lymphocytes at 6 days after infection, and HIV-1-treated astrocytes or combined TNF-α- and HIV-1-treated astrocytes and lymphocytes at 48 h after treatment. Uninfected culture samples were used as controls.

Briefly, undiluted culture fluids or diluted (1:2) CSF samples (50 μl) were added to ELISA plate wells containing analyte beads in duplicate followed by incubation for 30 minutes at RT. After washing, antibody-biotin reporter was added and incubated for 30 minutes at RT. After washing, streptavidin-phycoerythrin was added and incubated for 10 minutes at RT. Beadlyte readings were taken in a Luminex reader (Austin, TX) and calculated using Bioplex software (Bio-Rad). The sensitivity was > 5 pg/ml for each analyte. Quantification of signals was based on the standard beads run in parallel to the test.

TNF-α and CXCL10 were estimated in supernatants from uninfected or HIV-1-infected macrophages and HFA using TNF-α and CXCL10 ELISA kits (BD Biosciences) as per the manufacturer’s protocol. HFA were seeded in 24-well plates at a density of 0.3 × 10^5^ cells/well and kept for 2 days. On the third day, cells were treated with HIV-1 (250 ng/ml p24) and TNF-α (10 ng/ml) either alone or in combination. In parallel, cells were treated with IFN-γ (100 ng/ml) as a positive control. At 48 h after treatment, culture supernatants were harvested for CXCL10 and TNF-α.

### Cytotoxicity assay

Neuronal and astrocyte toxicity assays were done in 48- or 96-well cell culture plates using cell viability QuantiBlue and lactate dehydrogenase (LDH) assays [58,59] or flour-Jade C staining (Millipore). For direct toxicity studies, we used equal concentrations (250 ng/ml p24 antigen) of X4-tropic HIV-1 NL4-3, mutant-HIV-1 defective in integrase function (ΔINT NL4-3), and R5-tropic NLYU2 virus particles, for which viral DNAs were packaged in 293T cells as described earlier [45,52]. In parallel, H_2_O_2_ was used as a positive control; medium was used as a negative control. Cells were cultured overnight in optiMEM (HFN) or DMEM (HFA) and given different treatments, as indicated in the figures. The LDH assay was done 48 h after treatment of HFN and HFA. Briefly, total LDH activity was calculated by lysing the cells with 1% Triton-X for 20 minutes at 37°C. Results were expressed as percent LDH release calculated as follows:

$$\%\;\text{LDH}=\left(\text{OD}\left(\text{test}\right)/\text{OD}\left(\text{total}\right)\right)\times 100$$

Cell viability was quantified using QuantiBlue assay (Bioassay Systems) [45], which uses a redox nonfluorescent dye, resuzurin; this is reduced to a highly fluorescent product by metabolically active cells. Briefly, at the end of treatments, 10 μl of dye was added to each culture well containing 100 μl of medium and incubated for 3 h at 37°C. Fluorescence in the cells was measured using Optilux black plates (Falcon) at an excitation of 530 nm and emission at 590 nm in a fluorescence plate reader (BioTek).

In microscopic quantification, after staining with propidium iodide (PI) (10 μg/ml) and counterstaining for nuclei with Hoechst (1 μg/ml) for 10 minutes at 37°C, PI uptake was estimated in cultured cells. Cells showing red puncta were counted in five low-power fields (lpf); mean number of PI-stained cells were calculated.

JC1 staining for mitochondrial membrane potential was performed as described earlier [51]. In normal cells mitochondrial membrane potential (δΨ) remains high. Membrane permeable JC1 staining results in formation of red colored JC1 aggregates (590 nm emission) while during mitochondrial damage JC1 exists in monomer form that gives green color emission (538 nm). The ratio of 538 and 590 nm gives an estimate of mitochondrial membrane potential (mitochondrial health). At 6 h after treatment either with HIV alone or in combination with cytokines, HFA were stained with JC1 dye. Briefly, 10 ug/ml JC1 (Sigma) was diluted in complete medium and added to the cells. The δΨ was assessed following incubation for 20 minutes in the dark at 37°C. Cellular fluorescence was read on a florimeter (BioTek) following excitation at 488 nm. Mitochondrial-δψ was read as the ratio of emission at 538 nm and 590 nm. Potassium ionophore, valinomycin (100 nM) was used as a positive control.

### RNA extraction, cDNA synthesis, and real-time PCR

Total RNA was extracted from HFA using Trizol reagent (Gibco-BRL). This was followed by treatment with 10 units of RNase- free DNase-1 (Qiagen). RNA was further purified using RNAeasy plus mini columns (Qiagen) [51] and quantified by measuring absorbance at OD_280_. cDNA was synthesized from 6 μg RNA using random hexamers and Superscript II^TM^ reverse transcriptase (Invitrogen) in a total volume of 60 μl at 42°C for 1 h [51]. Each real-time PCR reaction was assembled using 1 μl of cDNA template scaled to a 30-μl reaction (triplicate), Sybr green master mix (Bio-Rad) with primer sets for gag gene, gag-f (5^′^-tcaatgaggaagctgcagaatggg-3^′^), gag-r (5^′^-tggttctctcatctggcctggtgc-3^′^); tat gene, tat-f (5^′^-gaagcatccaggaagtcagcc-3^′^) tat-r (5^′^-acaaacttggcaatgaaagcaacac-3^′^); nef gene, nef-f (5^′^-cctgcatggaatggatgaccctgag-3^′^) and nef-r (5^′^-gggccacgtgatgaaatgctaggcg-3^′^). The signals were normalized with glyceraldehyde-3-phosphate dehydrogenase (GAPDH) using primers GAPDH-f (5^′^-catcagcaatgcctcctgcacc-3^′^) and GAPDH-r (5^′^-gtgctcagtgtagcccaggatg-3^′^). Serial dilutions of HIV-1 pNL4-3 plasmid DNA as a template were used to calculate primer efficiencies of all primer sets. In parallel, negative control in which no DNA polymerase was present and in another control no cDNA template was present, were used. Reactions were run on a CFX96 real-time PCR system (Bio-RAD). Data were collected and analyzed using Bio-RAD CFX Manager Software v 1.1. Authentic PCR amplification was validated by examining whether any non-specific amplification occurs from RNA. Ct values were calculated for each gene and normalized relative to GAPDH expression. Fold expression from untreated control or ΔΔCt was calculated using the Paffafl method as reported earlier [60].

### Immunofluorescence and flow cytometry

HFA and HFN were seeded on 8-chambered slides (LabTek) coated with poly-D-lysine (20 μg/ml) (Sigma), then cultured for 4 days in complete medium. After various treatments, cells were processed for immunofluorescence as described earlier [52,53]. Briefly, cells were fixed with 2% paraformaldehyde in buffered saline for 15 minutes. After washing, cells were permeabilized with 0.1% Triton X containing 0.01 M glycine in PBS for 11 minutes, stained overnight with monoclonal anti-GFAP (1:400) or MAP-2 (1/400) (both from Sigma), and labeled with anti-mouse alexa-569 conjugate antibody (1:500). For CXCR3 or CXCR4 receptors, cultures were immunostained overnight at 4°C using human anti-CXCR3 (1:400) (R&D) and labeled with anti-mouse Alexa 488 (1:500) for 1 h at RT, then stained overnight with human anti-CXCR4-PE conjugated antibody (1 μg/ml) at 4°C (eBiosciences). For expression of IFN-γ, astrocytes treated either alone with TNF-α (10 ng/ml) or in combination with HIV-1 (250 ng/ml) for 48 h were immunostained with anti human IFN-γ Alexa 488 (eBiosciences) at 4°C overnight. After IFN-γ immunostaining, astrocytes were co-immunostained for GFAP marker as described above. To stain damaged neurons, immunostained cells were treated with 0.006% KMnO_4_ for 5 minutes, then stained with 0.0001% Fluoro-Jade C (Millipore). Cells were counterstained for nuclei with 1 μg/ml Hoechst (Sigma) and counted for cell death using a fluorescence microscope (Nikon). Images were captured with a digital camera (Nikon).

CXCR4 expression on HFA and HFN were monitored using flowcytometry. HFA and HFN were grown in 6-well plates for a week. After mild trypsinization for 60 sec to avoid removal of surface receptors, trypsin was neutralized with 10% FBS and cells were washed. The cells were resuspended in 1% BSA containing PBS, then stained using appropriate antibodies conjugated with PE and the isotype control antibody for 1 h at 4°C; 100,000 cells per sample were acquired and analyzed using flow cytometry (Beckman Coulter).

### Cell migration assay

Using a modified Boyden chamber (NeuroProbe) method, we monitored the chemotactic activity of supernatants collected from astrocytes treated with HIV-1, HIV-1 plus bryostatin, or supernatants from HIV-1 infected macrophages. Briefly, 30 μl of each supernatant in quadruplicate was used in the lower chamber, separated by a polycarbonate membrane (5 μm pore size) in a 96-well culture plate. Activated PBMCs were washed twice in optiMEM. We seeded 50,000 cells per well in a 50-μl volume on top of the membrane. We incubated the culture plate at 37°C for 90 minutes. Cells that had migrated across the membrane in the lower chamber were recovered by centrifugation at 1,000 g for 2 minutes and transferred to BD-Optilux black plates. In parallel, 2-fold serial dilutions of PBMCs were used to make a standard curve. To estimate migrated cells across the membrane, we established a plate-based staining procedure, staining harvested cells with 0.2 μM ethidium bromide in 10 mM Tris chloride and 0.1% Triton × 100 for 10 minutes. Fluorescence was read at an excitation of 530/30 nm and emission at 590/30 nm using a Synergy2 plate reader (BioTek). The number of migrated cells was determined by standard curve.

### Statistics

Results are represented as mean ± standard error of the mean (SEM) for each bar set plotted using Sigma plot v8.0 with associated *P*-values for each treatment group compared to its control. Statistical analysis was done using Origin 6.1 software. Significance between two groups was calculated using the two-tailed Student’s *t*-test. *P* < 0.05 was considered significant. For patients’ CSF data, results were further validated using the nonparametric two-tailed Mann-Whitney *U* test.

## Results

### Signatory proinflammatory chemokines and cytokines in HIV-1-infected demented patients

**Figure 1:**
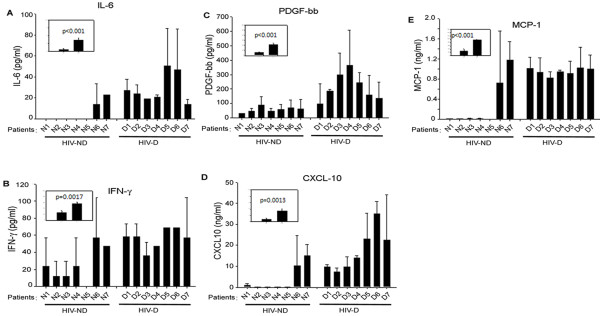
**Analysis of cytokines and chemokines by Bioplex array on CSF samples (A-C) cytokines and (D, E), chemokines in CSF samples from HIV-D and HIV-ND patients.** CSF samples were diluted 1:2 and 50 μl of each sample was used in the Bioplex array. Insets show mean elevated levels of (**A**) IL-6 (5.5- fold), (**B**) IFN-γ (2.2- fold), (**C**) PDGF-β (3.7- fold), (**D**) CXCL10 (4.6- fold), and (**E**) MCP-1 (3.4- fold) in CSF from HIV-D patients as compared to levels in HIV-ND patients. CSF, cerebrospinal fluid; HIV-D, HIV-demented; HIV-ND, HIV-nondemented; PGDF, platelet-derived growth factor; MCP, monocyte chemotactic protein.

We investigated the proinflammatory response in the CSF of HIV-D and HIV-ND patients using Bioplex array. CSF samples were profiled for cytokines and chemokines using a custom-made 10-plex Bioplex array. That analysis showed that five signatory chemokines/cytokines (IL-6, IFN-γ, PDGF-bb, CXCL-10, and MCP-1) were upregulated in HIV-D patients, but not HIV-ND patients (Figure 1). Other proinflammatory molecules tested (IL-8, IL-1β, IL-17, MIP-1α) were expressed at basal levels in both groups (data not shown). In the CSF of HIV-D patients as compared to HIV-ND patients, signaling IL-6, IFN-γ and PDGF-bb cytokines were respectively upregulated 5.5-fold (28.8 ± 5.4 pg/ml), 2.2-fold (56.4 ± 5.0 pg/ml), and 2.4-fold (212.7 ± 36 pg/ml) (Figure 1A-C, inset). Similarly CXCL10 and MCP-1 chemokines were increased 4.6-fold (17.3 ± 3.2 ng/ml) and 3.4-fold (949 ± 52 ng/ml) (Figure 1D, E) in HIV-D. To elucidate the source of the proinflammatory cytokines/chemokines, we investigated HIV-1 infection in astrocytes, lymphocytes, and macrophages, given their roles in neurological complications. Of note, TNF-α was undetectable in CSF but was found in HIV-1 infected macrophages.

### HIV-1 infection in macrophages and lymphocytes provokes CXCL10, MCP-1, and TNF-α

**Figure 2:**
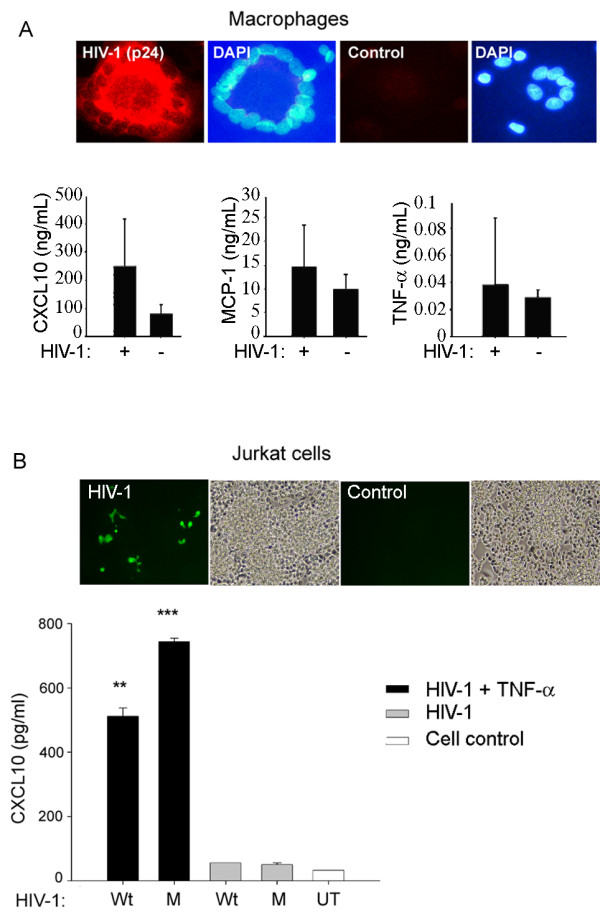
**HIV-1-infected macrophages and lymphocytes induced CXCL10, MCP-1, and TNF-α.** (**A**) Primary human macrophages were mock-infected or infected with HIV-1 B-aL (100 ng/ml p24). Supernatants collected on the day 14 and 16 post-infection were analyzed for cytokine/chemokine profile. Upper panel shows immunostaining for viral p24 antigen (red) in HIV-1-infected but not -uninfected (control) macrophages at 16 days post infection. Nuclei were stained with DAPI (blue). (**B**) Jurkat cells were co-treated with TNF-α (10 ng/ml) and either wild-type HIV-1 NLENG1 (wt) or integrase-defective mutant HIV-1 (M) particles for 48 h. Supernatants collected at 48 h after treatment were analyzed for CXCL10 production by ELISA. Upper panel shows GFP expression in HIV-1-infected but not uninfected Jurkat cells. DAPI, 4',6-diamidino-2-phenylindole; MCP, monocyte chemotactic protein; GFP, green fluorescent protein.

Primary human macrophages and lymphocytes were either infected with M-tropic HIV-1 (Ba-L strain or NLENYU2), T-tropic NLENG1, or left uninfected. Culture supernatants from infected and uninfected macrophages were collected on day 14 and 16 after infection and from lymphocytes on day 4. We monitored infection in lymphocytes by fluorescence microscopy and in macrophages by immunostaining for p24. At day 16, immunostaining demonstrated high expression of viral p24 antigen in HIV-1 infected, but not uninfected macrophages (Figure 2A, upper panel). Using viral p24 antigen ELISA, a viral peak was detected between day 14 and 16 after infection, but not in controls (data not shown). Interestingly, the proinflammatory response of supernatants from HIV-1-infected macrophages on day 16 after infection showed marked increases in CXCL10, MCP-1, and TNF-α by Bioplex array (Figure 2A, lower panels), while other cytokines and chemokines were not significantly changed (data not shown). CXCL10 and MCP-1 levels in HIV-1- infected macrophages were most prominent (Figure 2A).

Given the infiltration of T-cells, albeit less pronounced than monocytes or macrophages in HIV-infected brains, we further examined the proinflammatory response of lymphocytes to HIV-1 and TNF-α. HIV-1 infection as seen by GFP expression occurred in infected but not uninfected Jurkat cells after 48 h (Figure 2B, upper panel). At 48 h after treatment with either HIV-1 alone or in combination with TNF-α, Jurkat cells showed approximately 10-fold induction in CXCL10 levels as compared to HIV-1-infected or normal controls (Figure 2B). However, the proinflammatory response of Jurkat cells to combined HIV-1 and TNF-α treatment was much less pronounced than that in infected macrophages (Figure 2A and B). Overall, HIV-1 distinctly provoked increases in CXCL10 in macrophages, and lymphocytes.

### TNF-α and HIV-1 induce a proinflammatory response in astrocytes similar to that in HIV-D patients

Elevation of TNF-α, which is secreted from HIV-1-infected macrophages or microglia, has been widely shown to occur in response to hepatitis C virus (HCV) co-infection or methamphetamine abuse in HIV-1 infection settings [39,61-65]. In this study, TNF-α was undetectable in CSF samples, but was found in culture supernatants of HIV-1 infected macrophages by Bioplex array. We investigated the possible role of TNF-α, either alone or in combination with HIV-1, in provoking a proinflammatory cascade in astrocytes.

**Figure 3:**
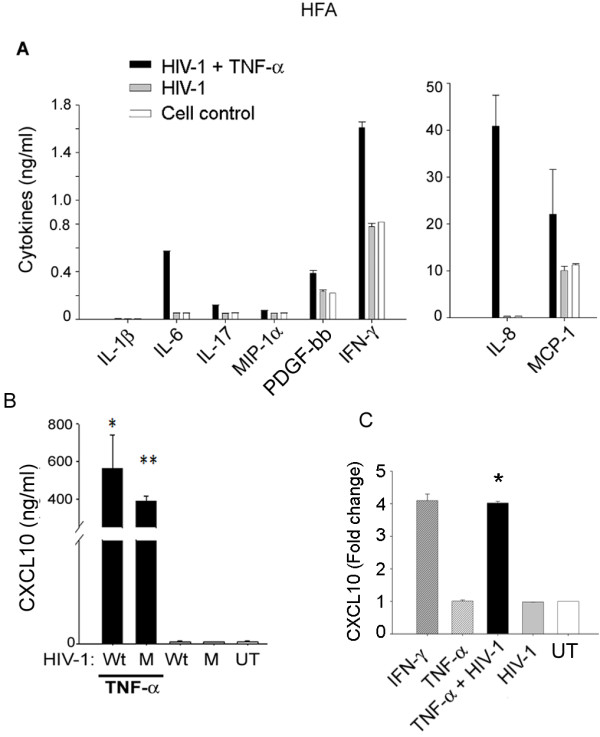
**HIV-1 and TNF-α induced CXCL10 production in astrocytes.** (**A**) HFA were treated with TNF-α (10 ng/ml) in combination with equal concentrations (250 ng/ml p24) of either wild-type HIV-1 NL4-3 (wt) or integrase-defective mutant HIV-1 (M) for 24 h. Cultures were washed once and replaced with fresh medium. Supernatants collected at 48 h after treatment were analyzed for cytokines/chemokines by Bioplex array. (**A**) IFN-γ, IL-8, MCP-1, PDGF, and (**B**) CXCL10 were elevated in culture supernatants collected from TNF-α- and HIV-1-treated HFA. (**B-C**) CXCL10 production from HFA was confirmed by quantitative ELISA. HFA, human fetal astrocytes; MCP, monocyte chemotactic protein; PDGF, platelet-derived growth factor.

We treated HFA with HIV-1 (250 ng/ml p24), TNF-α (10 ng/ml), or a combination of these two, for 48 h, then monitored the cytokine or chemokine response using a Bioplex array. Given the presence of M-tropic and T-tropic viruses in the brains of HIV-1 patients [2], we tested stocks of different doses of M-tropic and T-tropic HIV-1 prepared in HEK-293 cells (free of proinflammatory products). We found that a concentration of 250 ng/ml was effective in toxicity studies on HFA (unpublished), and used this in further studies. Interestingly, co-treatment with HIV-1 and TNF-α showed a robust proinflammatory response similar to that observed in HIV-D patients, but TNF-α alone was ineffective (Figure 3A). Compared to controls, the levels of cytokines or chemokines were upregulated as follows; IL-1β, 2-fold; IL-6, 11.2-fold; IL-17, 2.2-fold; MIP-1α, 1.5-fold; PDGF-bb, 1.4-fold; and IFN-γ, 2-fold (Figure 3A, left panel). In astrocytes, co-treatment with HIV-1 and TNF-α induced IL-8 and MCP-1 (Figure 3A right panel) similar to IFN-γ. Remarkably, CXCL10 was profoundly upregulated (> 1,000-fold) in astrocytes after co-treatment with TNF-α and either wild-type (wt) or mutant (defective in integrase function) HIV-1 particles (Figure 3B, C). This finding, validated by ELISA, suggests that HIV-1 without replication can induce CXCL10 production in bystander cells (Figure 3C). Treatment with IFN-γ, a known inducer of CXCL10, was used as a positive control. Interestingly, HIV-1 and TNF-α treated astrocytes showed synergistic upregulation of CXCL10 production similar to that of IFN-γ (Figure 3C). Overall, TNF-α in combination with HIV-1 distinctly provoked an increase in CXCL10 in astrocytes. To further investigate the significance of TNF-α and HIV-1, we studied their effect on neurons and astrocytes.

### TNF-α synergizes HIV-1-induced toxicity in astrocytes but not in neurons

**Figure 4:**
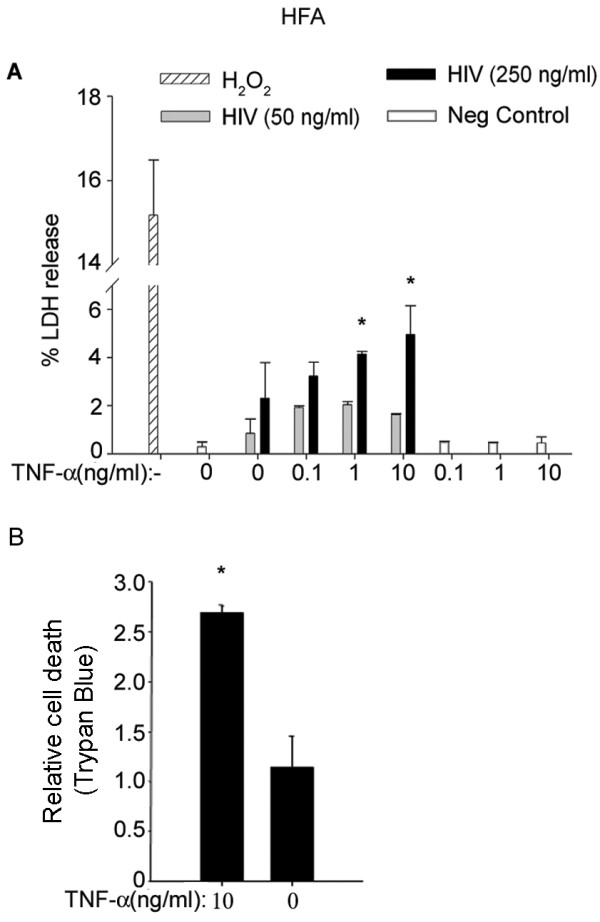
**TNF-α synergized HIV-1-induced toxicity in astrocytes.** (**A**) TNF-α with HIV-1 increased toxicity on astrocytes (HFA). LDH release was estimated in culture supernatants from HFA after treatment with increasing concentrations of TNF-α (0–10 ng/ml) and HIV-1 NL4-3 (50 ng/ml, 250 ng/ml p24 concentrations) or TNF-α alone for 48 h. Controls were left untreated (negative control). H_2_O_2_ was used as a positive control (*P* < 0.05). (**B**) HFA were treated with HIV-1 alone (250 ng/ml) or in combination with TNF-α (10 ng/ml). At 48 h after treatment, cell death was monitored by Trypan blue dye exclusion. Untreated HFA served as a negative control. Results were plotted as mean ± standard error of the mean (SEM) after counting 20 high-power fields for each treatment (n = 3). HFA, human fetal astrocytes; LDH, lactate dehyrdrogenase.

To study the effect of TNF-α in brain injury, we investigated its impact on HIV-1-induced glial toxicity. Having found that both M-tropic and T-tropic HIV-1 were equally cytotoxic to astrocytes (Mehla and Chauhan, 2011, unpublished), we monitored the toxicity profile of HFA at 48 h after treatment with HIV-1 NL4-3 virus particles (50 and 250 ng/ml p24) and TNF-α (0.1-10 ng/ml). Initially, we used an LDH release assay on culture supernatants to monitor cytotoxicity, then later confirmed our results by propidium iodide uptake, Trypan blue, or MTT assay. Interestingly, co-treatment of astrocytes with TNF-α and HIV-1 dose-dependently caused exaggerated toxicity in LDH release (Figure 4A) and Trypan blue uptake assays (Figure 4B).

**Figure 5:**
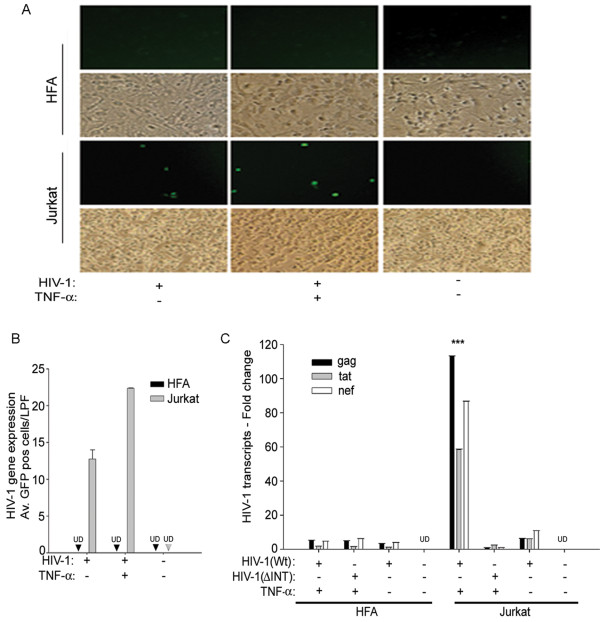
**Effect of TNF-α on HIV-1 transcript expression in infected astrocytes.** HFA and Jurkat cells (positive control) were infected with HIV-1 NLENG1 (50 ng/ml) (YFP-expressing HIV-1 to track infection in live cells) and treated with TNF-α (10 ng/ml) or left untreated. (**A**) HIV-1 infection was assessed by the number of GFP-expressing cells. Representative pictures of Jurkat and HFA showing GFP-positive cells. (**B**) Quantification of HIV-1 infection was done by counting GFP-positive cells in 10 random fields and plotted as mean ± standard error of the mean (SEM). (**C**) HFA and Jurkat cells were treated with TNF-α (10 ng/ml) in combination with either HIV-1 wt or HIV-1 integrase- defective mutant (ΔINT) as a negative control for viral replication. Cells were harvested after 48 h of treatment for total RNA and monitored for early and late viral mRNA transcripts (*tat*, *nef*, and *gag*) using reverse-transcription coupled real-time PCR. UD = undetectable (n = 2). HFA, human fetal astrocytes; GFP, green fluorescent protein; wt, wild type.

TNF-α activates HIV-1 transcription via activation of NF-kB in lymphocytes and macrophages, and consequently provokes a proinflammatory response [66-68]. Thus, TNF-α may increase viral protein synthesis and consequently exaggerate the toxic response by astrocytes, resulting in neurotoxicity. To rule out exaggerated toxicity, if any, by increased viral replication in astrocytes as compared to that in neurons, we infected HFA with HIV-NLENG1 virus, which expresses GFP [45,56,60]. This allows monitoring of HIV-1 gene expression in real time. HFA, in parallel with Jurkat cells (positive control), were infected with HIV-1 and treated with TNF-α; 48 h later we monitored GFP expression. TNF-α treatment either pre- or post-infection did not cause an increase in GFP expression and remained undetectable in HIV-1 infected HFA, while Jurkat cells showed substantial increases in viral infection as demonstrated by GFP positivity (Figure 5A, B).

**Figure 6:**
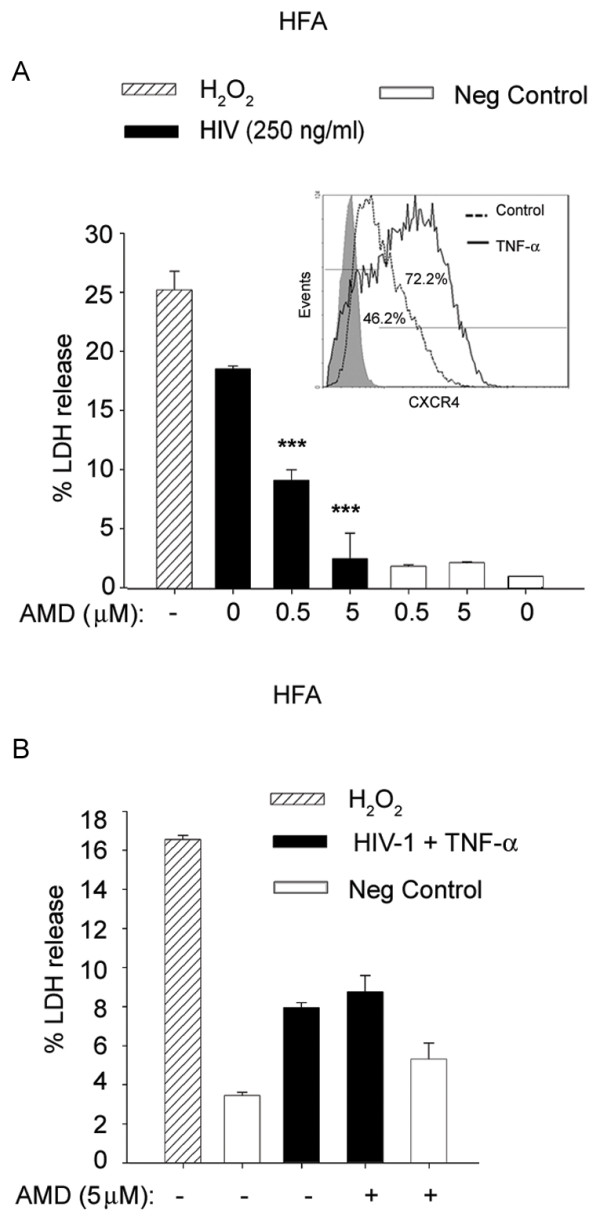
**CXCR4 blocker attenuated HIV-1 but not combined TNF-α and HIV-1-induced astrocyte toxicity.** (**A**) HFA pretreated with CXCR4 blocker (AMD3100) (0.5 or 5.0 μM) were given HIV-1 treatment; 48 h later, LDH release was estimated in cell supernatants (*P* < 0.05). Inset shows overlay histogram of CXCR4 receptor expression in HFA after treatment with TNF-α for 16 h analyzed by flow cytometry, with the percentage of cell population shown relative to isotype control. (**B**) HFA were pretreated with AMD3100 (CXCR4 blocker) for 30 minutes at the indicated concentrations, then treated with a combination of HIV-1 (250 ng/ml) and TNF-α (10 ng/ml) and monitored for LDH release as before. HFA, human fetal astrocytes; LDH, lactate dehyrdrogenase.

To determine whether HIV-1 gene expression occurs in HFA without GFP expression, we co-treated HFA with HIV-1 and TNF-α for 48 h and monitored viral early (*tat* and *nef*)- and late (*gag*)-mRNA transcripts using reverse-transcription-coupled real-time PCR. In parallel, we used lymphocytes as positive controls. Unlike HIV-1 susceptible lymphocytic cells, viral transcripts in HFA remained at undetectable or basal levels after treatment with TNF-α (Figure 5C), indicating that TNF-α-synergized HIV-1-induced toxicity in astrocytes was independent of productive HIV-1 infection, rather than being directly induced by viral particles. In parallel, we used equal concentrations of HIV-1 mutant virus that was defective in integrase function (incapable of productive infection, ΔINT) as an additional control (Figure 5C). Mutant HIV-1 gave results similar to those obtained with wt virus, thus reinstating the viral envelope-mediated signaling event. To confirm this, we used AMD3100, a CXCR4 receptor blocker, to reverse HIV-1-induced toxicity. HFA showed high expression of CXCR4 (Figure 6A, inset) and AMD3100 dose dependently attenuated HIV-1-induced toxicity (Figure 6A). Intriguingly, AMD3100 did not attenuate HIV-1+TNF-α-induced toxicity in HFA (Figure 6B), suggesting induction of a different toxic pathway in astrocytes.

**Figure 7:**
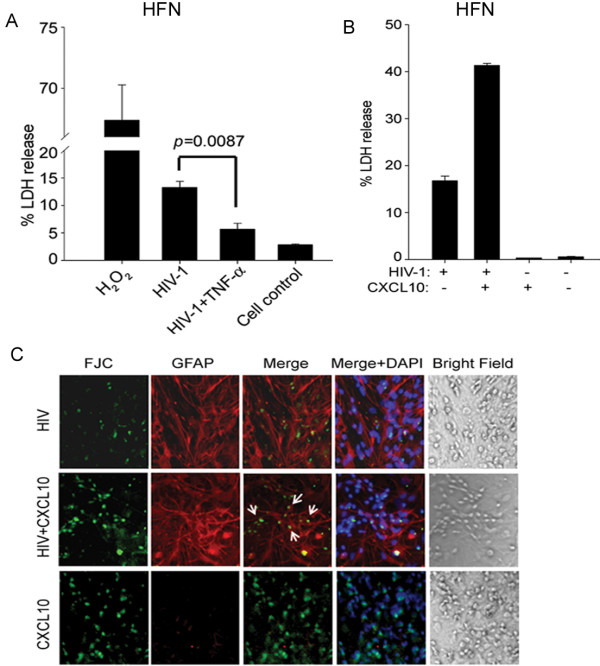
**CXCL10, but not TNF-α, synergized HIV-1-induced neuronal toxicity.** (**A**) LDH activity was estimated in supernatants from HFN treated with HIV-1 alone or in combination with TNF-α. H_2_O_2_ was used as a positive control. (**B**) Mixed neuronal cultures (HFN) were co-treated with HIV-1 (250 ng/ml p24 concentration) and CXCL10 for 24 h. LDH release was estimated on cell supernatants. (**C**) HIV-1 and CXCL10 co-treated HFN were immunostained for GFAP (red), then counterstained with FJC (green), which stains dead neurons. Nuclear staining was done with DAPI (blue). LDH, lactate dehyrdrogenase; HFN, human fetal neurons; GFAP, glial fibrillary acidic protein; FJC, fluoro-jade C; DAPI, 4',6-diamidino-2-phenylindole.

Subsequently, we tested the effect of HIV-1 particles in combination with TNF-α on primary HFN. The HFN cultures grown for two months were used in the experiments. Co-treatment of HFN with TNF-α and HIV-1 showed no enhanced toxicity and, indeed, suppressed neurotoxicity (Figure 7A), suggesting some intracellular neuroprotective programming that needs to be investigated further.

### HIV-1 with CXCL10, but not TNF-α, synergistically induced neuronal toxicity

It has been shown that elevated levels of CXCL10 and MCP-1 in the CSF of HIV-D patients are chemo-attractants for lymphocytes and monocytes or macrophages, resulting in an exaggerated neuroinflammatory response [8,14]. We found that HIV-1 and TNF-α together induced release of various chemokines, including CXCL10, from astrocytes and lymphocytes. Also, HIV-D CSF showed elevated CXCL10 levels. We investigated whether CXCL10 in combination with HIV-1 particles compromise the survival of neurons. Treatment of neurons with HIV-1 (250 ng/ml p24) and CXCL10 (100 ng/ml) for 48 h showed exaggerated toxicity in an LDH release assay (Figure 7B). We also did FJC staining for dying neurons and immunostaining for astrocytes using anti-GFAP. FJC stains degenerating neurons irrespective of the type of insult. After co-treatment of mixed neuronal cultures with CXCL10 and HIV-1, increased expression of GFAP positivity was coupled with neuronal damage (Figure 7C). This suggests that TNF-α and HIV-1 deregulate astrocyte function more severely than does HIV alone (Figure 2) and eventually compromises neuronal functions via CXCL10 (Figure 7B, C). However, TNF-α may also induce neuroprotection. A possibly similar neuroprotective effect was observed in our mixed neuronal cultures using TNF-α and HIV-1 insult (Figure 7A). Nottet *et al*. [13] found that astrocytes dampen TNF-α-induced neurotoxicity when co-cultured with HIV-1-infected macrophages and neurons. However, our neuronal cultures lacked microglia and macrophage phenotypes and may not have reflected all the consequences of the *in vivo* situation. Together, our results concluded that HIV-1 in combination with CXCL10, but not TNF-α, induces neuronal toxicity.

### Ablation of CXCL10 and HIV-1-induced neurotoxicity by the mitogen-activated protein kinase inhibitor and PKC activator

**Figure 8:**
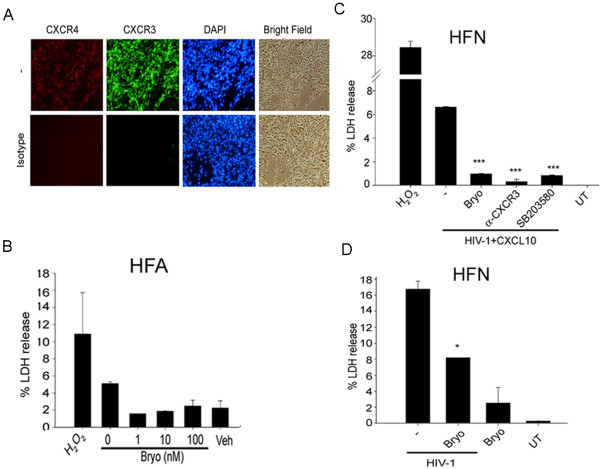
**PKC activator (bryostatin) and MAPK inhibitor protected against CXCL10- and HIV-1-induced neurotoxicity.** (**A**) HFN cultures were immunostained for CXCR3 (green) and CXCR4-PE (red), then counterstained with DAPI (n = 3). (**B**) Dose response of bryostatin for toxicity; HFA cultures were treated with increasing concentrations of bryostatin (0 to 100 nM). LDH was estimated in supernatants 48 h post-treatment. (**C-D**) HFN were pretreated with bryostatin (25 nM), CXCR3 blocking monoclonal antibody (5 μg/ml), or MAPK inhibitor SB203580 (5 μM), then insulted with a combination of (**C**) HIV-1 and CXCL10 or (**D**) HIV-1 alone. At 24 h post-treatment, LDH activity was monitored in supernatants. H_2_O_2_ was used as a positive control (n = 2). PKC, protein kinase C; MAPK, mitogen-activated protein kinase; HFN, human fetal neurons; DAPI, 4',6-diamidino-2-phenylindole; HFA, human fetal astrocytes; LDH, lactate dehydrogenase.

To investigate the mechanism of CXCL10- and HIV-1-induced neurotoxicity, we monitored HFN for expression of CXCR3 and CXCR4, which has been reported to occur in various cells, including activated lymphocytes, monocytes, and endothelial cells [37,69]. HFN showed expression of CXCR4, a co-receptor for HIV-1, and of CXCR3, a receptor for CXCL10 (Figure 8A). CXCR4 is the receptor that engages HIV-1 gp120 and Tat proteins on the cell surface, resulting in transduction of intracellular signals. CXCR4 alone is not responsible for transducing the neurotoxic signal, but may trigger a proinflammatory response. Binding of CXCL10 to CXCR3 receptors transduces the signaling cascade by activating the mitogen-activated protein kinase (MAPK) pathway [70]. To investigate the involvement of CXCL10-CXCR3 signaling in HIV-1-induced neuronal toxicity, we pretreated neurons with 5 μg/ml of CXCR3 blocking antibody (R&D systems) or MAPK p38 inhibitor (SB203580) and monitored neurotoxicity by LDH release 24 h later. To investigate the role of the CXCR4 receptor in HIV-1-induced neurotoxicity, we targeted the PKC pathway (bryostatin), which we and others have shown to be involved in CXCR4 regulation [43-45]. Protein kinase activation by bryostatin has been shown to decrease expression of CXCR4 on lymphocytes and result in reduced HIV-1 infection [45]. To find the working nontoxic concentration of bryostatin, we initially did a dose response test on HFA using different concentrations of bryostatin (1 to 100 nM). No toxic effects were seen by LDH assay (Figure 8B). Blocking either CXCR3 receptors or MAPK signaling annihilated HIV-1 and CXCL10-induced neurotoxicity (Figure 8C) indicating involvement of the CXCL10-CXCR3 pathway. Treatment of neuronal cultures at a 25 nM concentration of bryostatin profoundly suppressed both HIV-1- and CXCL10 + HIV-1-induced neurotoxicity (Figure 8C, D).

Thus, to determine whether CXCR4 and CXCR3 receptors are involved in transducing toxicity signals, we immunostained mixed neuronal cultures for GFAP, then stained them with FJC staining for neuronal death after combined HIV-1 and CXCL10 insult (Figures 7, 8). HIV-1-treated mixed neuronal cultures showed enhanced expression of astrocytic activation marker; in combination with CXCL10, it showed increased neuronal damage (Figures 7, 8). These results indicate that CXCL10-CXCR3 intersects with the HIV-CXCR4 pathway in neurons to enhance toxicity. Thus far, blocking either MAPK signaling or CXCR3 and CXCR4 receptors resulted in neuroprotection against HIV-1 or combined insult with HIV-1 and CXCL10, suggesting that the PKC activator bryostatin has therapeutic potential (Figure 8C, D).

### Bryostatin suppressed CXCL10 and HIV-1 induced chemotaxis of PBMCs

**Figure 9:**
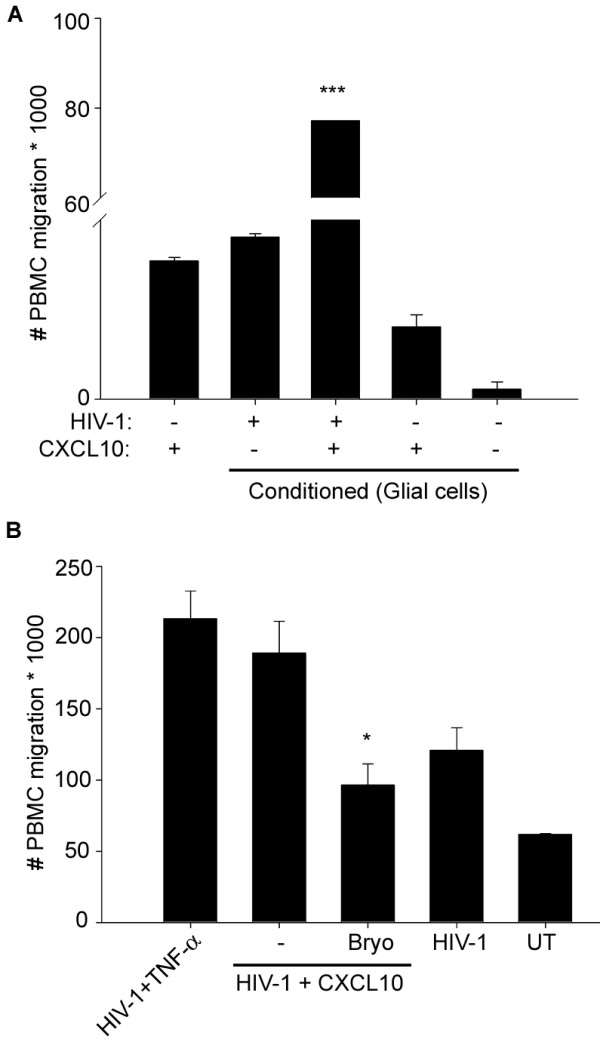
**PKC activator inhibited HIV-1- and CXCL10- induced chemotaxis of PBMC.** Activated PBMC were isolated from human blood donors and cultured in PHA and IL-2 for 2 days. Chemotaxis of PBMCs by culture supernatants collected from HIV-1 and CXCL10-treated HFA were done using a modified Boyden chamber. (**A**) Culture supernatants from HFA after 48 h of treatment with HIV-1 or a combination of HIV and CXCL10 were used in chemotaxis assays. CXCL10 (100 ng/ml) was used as a positive control (first bar). (**B**) HFA were pretreated with bryostatin (25 nM) for 30 minutes, then treated with a combination of HIV-1 (250 ng/ml p24) and CXCL10 (10 ng/ml) for 48 h. Culture supernatants were used in chemotaxis of PBMC. In parallel, supernatants from HFN cultures treated with a combination of HIV-1 and TNF-α were also used (*P* < 0.05) (n = 2). PKC, protein kinase C; PBMC, peripheral blood mononuclear cells; HFA, human fetal astrocytes; HFN, human fetal neurons.

CXCR3 expression on neurons in HIV-1-infected brains has been reported [71,72]. Moreover, induction of CXCL10 in HIV-1-infected patients with neurological complications has been demonstrated [7,9,33,35,71]. Given that migration of monocytes or macrophages and lymphocytes occurs in the brains of HIV-1-infected patients and results in neuroinflammation and neurotoxicity, we investigated whether CXCL-10 and HIV-1 confer any effect on migration of PBMC. To examine migration, we co-treated astrocytes with HIV-1 and CXCL10 in serum-free medium for 48 h. In parallel, we co-treated mixed neuronal cultures for 24 h with HIV-1 and CXCL10 or left them untreated (medium control). Cultures were washed to deplete residual CXCL-10 and HIV-1 particles and fresh medium was added. The conditioned supernatants were collected 48 h later and monitored for chemotactic potential for activated PBMC in a migration assay using a modified Boyden chamber (NeuroProbe). In parallel, we used CXCL-10 (100 ng/ml) or MCP-1 (50 ng/ml) as positive controls and mock-treated supernatants from HFA or HFN as negative controls. The supernatants from CXCL10 and HIV-1-co-treated HFA or HFN cultures showed profound increases in activated PBMC migration (Figure 9A), indicating that proinflammatory products, including CXCL10, are crucial in chemotaxis.

Given that bryostatin protected neurons against HIV-1 and CXCL10 toxicity, we investigated whether this PKC activator confers an inhibitory effect on CXCL10-induced chemotaxis. We co-treated HFA with HIV-1 and CXCL10, then with either bryostatin (25 nM) or vehicle control (dimethyl sulfoxide, DMSO). The supernatants, which were free of added HIV+CXCL-10 and bryostatin, were collected 48 h later and used in chemotaxis assays. The supernatants from bryostatin-treated CXCL10 and HIV-1 conditioned HFA, but not those from CXCL10 and HIV-1 conditioned supernatants, showed profound reductions in PBMC chemotaxis (Figure 9B), suggesting that bryostatin has significant therapeutic potential against HIV-1-induced neurotoxicity and chemotaxis. This result also made it clear that the bryostatin-mediated effect occurs by regulating cytokines and chemokines in astrocytes, but not directly at PBMC levels, since bryostatin was not present during the migration assays. Detailed investigations to elucidate the mechanism of bryostatin in neuroprotection and chemotaxis are in progress.

## Discussion

Gliosis and infiltration of activated monocytes, macrophages and lymphocytes in the brain compartment are important causes of inflammation and neurodegeneration in chronic HIV-1-infected patients [9,73,74]. Gliosis is characterized by the release of several cytokines and chemokines from activated glial cells both as astrocytes and microglia. TNF-α and other proinflammatory cytokines in the CSF and brain tissues of HIV-D patients correlate with the severity of HIV-1 disease progression [9,14,25,75,76]. *In vivo* and *in vitro* studies, including this one, have shown CXCL10 production from astrocytes following expression of gp120 or treatment with combination of TNF-α and IFN-γ with HIV-1 [21]. Second to microglia and macrophages, astrocytes are thought to be a source of the proinflammatory response in HIV-1 infection [77-79]. Activated T-cells in the brain also participate in neuroinflammation. However, upon T-cell depletion in the late stages of HIV-1 infection, monocytes and macrophages predominate in the migrated cell population in HIV-1 infected brains.

Using a Bioplex array on CSF, signatory cytokines and chemokines were seen; CXCL10 and MCP-1 were characteristic in HIV-D patients. On further *in vitro* investigation of astrocytes, macrophages, and lymphocytes, we found that CXCL10 was induced by HIV-1 infection or treatment with TNF-α or IFN-γ, suggesting that myeloid and lymphoid cell populations are key players in CXCL10. HIV-1 infection in macrophages also induced TNF-α. Similar to our earlier observations [52], HIV-1 infection in astrocytes was not observed within 48 h. Irrespective of the lack of productive HIV-1 infection, co-treatment of astrocytes with TNF-α and HIV-1 particles for 48 h showed upregulation of CXCL10 expression. Induction of CXCL10 in astrocytes could be an indirect effect of upregulation of other proinflammatory products, including IFN-γ. However, induction of CXCL10 in astrocytes by HIV-1 and TNF-α could be independent of IFN-γ. Although secretion of IFN-γ by activated or HIV-1 infected T-cells has been reported, no evidence on astrocytes has been documented. Recently, investigations of reovirus-infected animal brains demonstrated expression of IFN-γ in astrocytes [80]. Astrocytes under ischemic conditions also showed extracellular but not intracellular IFN-γ expression [81]. Similarly, in our current study, astrocyte cultures, on co-treatment with TNF-α and HIV-1, showed extracellular (Figure 3A) but not intracellular IFN-γ expression (data not shown). Overestimation of IFN-γ expression could be due to variation in the multiplex assay. Intracellular absence of IFN-γ in our study could be a consequence of the antibody used for detection after cell fixation; this needs further investigation. An earlier study on transgenic mice expressing HIV-1 gp120 under GFAP promoter did not detect IFN-γ in astrocytes [21]. It can be argued that ectopic application of HIV particles to astrocytes provokes intracellular signaling that is different from intracellularly expressed HIV-1 gp120 protein.

CXCL10 signaling is important in other brain pathologies, including Alzheimer’s disease and multiple sclerosis [30]. Astrocytes expressing CXCL10 are commonly associated with senile plaques [26]. TNF-α and HIV-1 induced toxicity in HFA, but unexpectedly reduced toxicity in HFN cultures. In earlier reports [82-84], TNF-α-mediated neurotoxicity was dampened by the presence of astrocytes either neutralizing the effect or producing the anti-inflammatory product IL-10. In our mixed neuronal cultures, the presence of astrocytes in the absence of microglia or macrophages might have conferred effects similar to those in the preceding studies. Although there is limited evidence of TNF-α production in HIV-infected brains, it has been suggested that HIV-1 primes TNF-α production in the presence of lipopolysaccharide (LPS). In our Bioplex array on CSF samples, no evidence of secretory TNF-α production was seen. However, such evidence was seen in the supernatants from HIV-1-infected macrophages. Nevertheless, TNF-α production may occur in situations such as drug abuse and the co-occurrence of other opportunistic infections with HIV-1 [85].

**Figure 10:**
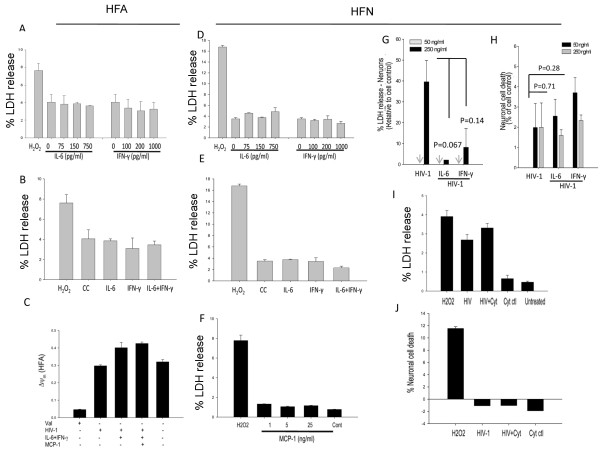
**Combined effect of IL-6, IFN-γ, and MCP-1 on neuroglial (HFN and HFA) damage.** (**A, B**) HFA and (**D, E**) HFN were treated with different doses of IL-6 (75 to 750 pg/ml) or IFN-γ (100 to 1,000 pg/ml) either alone or together (150 pg/ml IL-6 and 200 pg/ml IFN-γ). Toxicity was determined by LDH assay at 48 h after treatment. (***C***) IL-6 and IFN-γ, alone or in combination with MCP-1 (5 ng/ml), were tested in modulating mitochondrial membrane potential in HIV-1-treated astrocytes after 12 h of treatment. (**F**) Dose response of MCP-1 (1–25 ng/ml) in inducing neuroglial toxicity. (**G, H)**, Effect of IL-6 and IFN-γ in combinations with HIV-1 on the (**G**) toxicity profile (LDH) and (**H**) viability (MTT/QuantiBlue) of neurons at 48 h after treatment. Effects of IL-6, IFN-γ, and MCP-1 in combination with HIV-1 in modulating (**I**) toxicity (LDH) and (**J**) viability (QuantiBlue) at 48 h post-treatment in neurons. MCP, monocyte chemotactic protein; HFN, human fetal neurons; HFA, human fetal astrocytes; LDH, lactate dehydrogenase.

In addition to TNF-α, IL-6, which is also considered to be neurotoxic, is induced by HIV-1 gp120 [17]. However, IL-6 at the levels we detected in HIV-D patients’ CSF did not induce toxicity in HFA and HFN (Figure 10). Although IFN-γ and MCP-1 were also elevated in the CSF of HIV-D patients and *in vitro* in our studies, they failed, alone or in combination with IL-6, to induce neuroglial toxicity (Figure 10).

Interestingly, in an earlier study TNF-α-induced-CXCL10 expression stimulated HIV-1 replication in susceptible cells, including MDM and peripheral blood lymphocytes [86]. These cells migrate to the CNS compartment and contribute to elevated levels of inflammatory cytokines and viral proteins, which are HIV-1-derived toxic products. CSF from HIV-D patients, as compared to that from HIV-ND patients, showed a nearly 4-fold increase in CXCL10, indicating the importance of CXCL10 in the CNS. The CSF of two HIV-ND patients also had elevated CXCL10, indicating that these patients had ongoing viral activity that could progress to dementia. Thus, antiretroviral treatment is important in discouraging severe neurological complications; however, residual proinflammatory response in successfully treated HIV-infected patients nevertheless leads to minor cognitive impairment. In our study, CXCL10 was also induced at high levels in HIV-1-infected lymphocytes and macrophages and in HIV-1 + TNF-α-treated HFA. This indicates that neurons, during T-cell and macrophage infiltration in the brains of HAD patients, encounter localized concentrations of CXCL10 that exceed CSF levels. Moreover, a recent study has reported persistence of CXCL10 in the plasma of HIV-1 patients treated with antiviral therapy, suggesting that immune activation was still active during treatment [87]; this phenomenon may be more active in brains where antiviral drugs are less available. In addition to CXCL10, we found that MCP-1, a mediator of transmigration of monocytes or macrophages into the brain, was present in high levels both *in vivo* and *in vitro*.

Treatment of neurons with CXCL10 and HIV-1 had a synergistic effect on neuronal toxicity. Also, supernatants from CXCL10- and HIV-1-treated astrocytes had a profound effect on the migration of activated PBMC. Similarly, CXCL10 has been shown to cause neuronal damage in animal models [21,88,89]. CXCL10 is a potent chemokine that induces chemotaxis of immune cells (T-cells, monocytes/macrophages, NK cells, and plasmacytoid dendritic cells) [14]. In general, CXCL10 imparts its effects through CXCR3 receptors on target cells [22,90-92]. Chemokine CXCR3 receptors are abundantly distributed on neurons and neuronal processes in various HIV-1-susceptible cortical and subcortical brain regions [14,21,32]. In physiological conditions, chemokines are naturally regulated without inducing harmful consequences [93,94]. However, elevated CXCL10 expression in neurons in response to brain injury resulted in activation and migration of microglia to injury sites. After ablating CXCR3 (knockout mice), which is a receptor for CXCL10, axon trimming was impeded [95,96]. To corroborate that CXCL10 signaling had toxic effects on neurons, we demonstrated CXCR3 receptors in human neuronal cultures (Figure 8). Blocking the CXCR3 receptors on neurons abolished the neuronal toxicity induced by HIV-1 and CXCL10. Further, a combination of HIV-1 and CXCL10 showed selective toxicity in neurons; this toxicity was abrogated by treatment with MAPK inhibitor. In addition, neurotoxicity induced by HIV-1 and CXCL10 or by HIV-1 alone was profoundly decreased by treatment with the PKC activator, bryostatin. Treatment with bryostatin is reported to confer pleotropic effects on neuro-glial cells, and is currently being tested in a phase II clinical trial for treatment against Alzheimer’s disease ( http://www.brni.org/scientific_research/clinical_trials.aspx). Bryostatin has been shown to confer neuroprotection on various disease models, not only enhancing learning, but also promoting nerve growth-factor-dependent outgrowth of neurites via PKC-ε [49,97,98]. Earlier, we found [45] that bryostatin treatment suppressed the expression of CXCR4 in lymphocytes and ablated activation of NF-kB, findings that were similar to observations reported in an earlier study [99]. We have also reported that bryostatin inhibits HIV-1 infection independent of viral receptors. In the current study, bryostatin also decreased the chemotactic activity of supernatants collected from astrocytes after co-treatment with HIV-1 and CXCL10, indicating that it has an anti-inflammatory and neuroprotective role. Hence, it is reasonable to expect that treatment with bryostatin, which crosses the blood-brain barrier, can decrease the migration of lymphocytes and monocytes or macrophages into the brain by affecting the expression of chemokines and cytokines in the setting of HIV infection in the brain.

**Figure 11:**
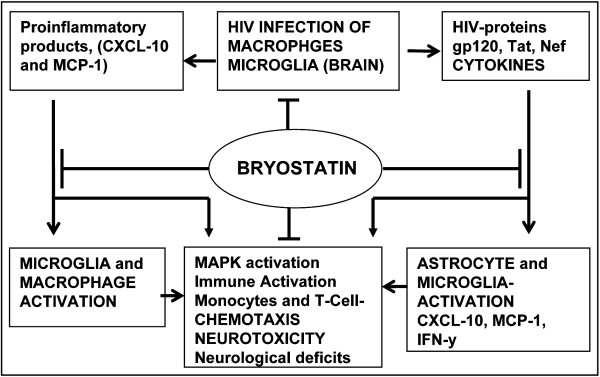
**Model depicting the interplay among HIV-1-induced proinflammatory products, neurotoxicity, and neuroprotection by bryostatin.** Proinflammatory products, including CXCL10, MCP-1 and TNF-α released from HIV-1-infected macrophages or microglia also activate astrocytes and microglia. CXCL10 in combination with HIV-1 induce neurotoxicity and chemotaxis of activated monocytes and T-cells. Bryostatin inhibits CXCL10- and HIV-1 induced neurotoxicity, and attenuates chemotaxis of monocytes and T-cells by regulating chemokines/cytokines expression in HIV-infected- or –uninfected- activated macrophages, microglia, astrocytes or T-cells. MCP, monocyte chemotactic protein.

Overall, we conclude not only that CXCL10 is upregulated in HIV-D patients, but that, in combination with HIV-1, can have a profound impact on neuroglial health in the neuro-HIV-1 infection setting (Figure 11). Bryostatin, a PKC activator, conferred neuroprotection against HIV-1 and CXCL10. Thus, we report for the first time that bryostatin can prevent neuronal damage and PBMC chemotaxis induced by HIV-1 and CXCL10 (Figures 9, 11). Although therapeutic targeting of chemokines in the brain may have adverse consequences, current findings and earlier published evidence suggest that CXCL10 could be a strong candidate in impeding neuroinflammation. Further understanding of the detailed molecular pathways induced by HIV-1 and CXCL10 in neurons and astrocytes will have great value in the design of novel neuroprotective therapies.

## Abbreviations

CNS: central nervous system; CSF: cerebrospinal fluid; DAPI: 4',6-diamidino-2-phenylindole; DMEM: Dubecco’s modified Eagle’s medium; DMSO: dimethyl sulfoxide; ELISA: enzyme-linked immunosorbent assay; FBS: fetal bovine serum; FCS: fetal calf serum; GFAP: glial fibrillary acidic protein; HAD: HIV-associated dementia; HFA: human fetal astrocytes; HFN: human fetal neurons; GFP: green fluorescent protein; HIV-D: HIV-demented; HIV-ND: HIV-nondemented; IP-10: 10-kDa IFN-γ-induced protein; LDH: lactate dehyrdrogenase; lpf: low-power fields; LPS: lipopolysaccharide; MAPK: mitogen-activated protein kinase; MCP: monocyte chemotactic protein; MDM: monocyte-derived macrophages; MIP: macrophage inflammatory protein; MNGC: multinucleated giant cells; PBMC: peripheral blood mononuclear cells; PBS: phosphate-buffered saline; PCR: polymerase chain reaction; PGDF: platelet-derived growth factor; PI: propidium iodide; PKC: protein kinase C; RT: room temperature; SDF: stromal-cell-derived factor; SEM: standard error of the mean; TNF: tumor necrosis factor; wt: wild-type.

## Competing interests

The authors declare no competing interests.

## Authors’ contributions

AC, and RM conceived and designed the experiments; AC, RM, and SBM performed the experiments; AC, and RM analyzed the data; AC, and MN contributed reagents; AC, and RM wrote the paper. All authors have read and approved the final version of the manuscript.
